# The Pepper Mitogen-Activated Protein Kinase CaMAPK7 Acts as a Positive Regulator in Response to *Ralstonia solanacearum* Infection

**DOI:** 10.3389/fmicb.2021.664926

**Published:** 2021-07-06

**Authors:** Lanping Shi, Kan Zhang, Linjing Xie, Mingxing Yang, Baixue Xie, Shuilin He, Zhiqin Liu

**Affiliations:** ^1^Fujian Provincial Key Laboratory of Applied Genetics, Fujian Agriculture and Forestry University, Fuzhou, China; ^2^College of Agriculture, Fujian Agriculture and Forestry University, Fuzhou, China

**Keywords:** *Capsicum annuum*, mitogen-activated protein kinase, *Ralstonia solanacearum*, WRKY transcription factor, pathogenicity

## Abstract

Mitogen-activated protein kinase (MAPK) pathways play a vital role in multiple plant processes, including growth, development, and stress signaling, but their involvement in response to *Ralstonia solanacearum* is poorly understood, particularly in pepper plants. Herein, *CaMAPK7* was identified from the pepper genome and functionally analyzed. The accumulations of *CaMAPK7* transcripts and promoter activities were both significantly induced in response to *R. solanacearum* strain FJC100301 infection, and exogenously applied phytohormones, including methyl jasmonate (MeJA), brassinolide (BR), salicylic acid (SA), and ethephon (ETN), were decreased by abscisic acid (ABA) treatment. Virus-induced gene silencing (VIGS) of *CaMAPK7* significantly enhanced the susceptibility of pepper plants to infection by *R. solanacearum* and downregulated the defense-related marker genes, including *CaDEF1*, *CaPO2*, *CaSAR82A*, and *CaWRKY40*. In contrast, the ectopic overexpression of *CaMAPK7* in transgenic tobacco enhanced resistance to *R. solanacearum* and upregulated the defense-associated marker genes, including *NtHSR201*, *NtHSR203*, *NtPR4*, *PR1a/c*, *NtPR1b*, *NtCAT1*, and *NtACC*. Furthermore, transient overexpression of *CaMAPK7* in pepper leaves triggered intensive hypersensitive response (HR)-like cell death, H_2_O_2_ accumulation, and enriched CaWRKY40 at the promoters of its target genes and drove their transcript accumulations, including *CaDEF1*, *CaPO2*, and *CaSAR82A*. Taken together, these data indicate that *R. solanacearum* infection induced the expression of *CaMAPK7*, which indirectly modifies the binding of CaWRKY40 to its downstream targets, including *CaDEF1*, *CaPO2*, and *CaSAR82A*, ultimately leading to the activation of pepper immunity against *R. solanacearum*. The protein that responds to CaMAPK7 in pepper plants should be isolated in the future to build a signaling bridge between CaMAPK7 and CaWRKY40.

## Introduction

As sessile organisms, plants are frequently subjected to unfavorable environmental challenges, including biotic and abiotic stresses, and have to adapt their metabolism, growth, and development accordingly (Berriri et al., 2012). Among the environmental challenges, attack by pathogens is a major plant stress that disturbs cellular homeostasis, leads to serious retardation of crop growth and development, and ultimately causes the dramatic loss of crop yield and quality (Ali et al., 2020a; Bargues-Ribera and Gokhale, 2020). To protect themselves against invasion by pathogens, plants have evolved a sophisticated and economical defense system. A well-established model of plant immunity is composed of two interconnected pathway layers, including pathogen-associated microbial patterns (PAMPs)-triggered immunity (PTI) and effector-triggered immunity (ETI) (Jones and Dangl, 2006). The recognition of PAMPs by membrane-targeted pattern recognition receptors (PRRs) in plants triggers an array of signaling pathways, such as calcium flux, the production of reactive oxygen species (ROS), and the activation of mitogen-activated protein kinases (MAPKs). These early events in turn activate the expression of downstream target proteins, such as transcription factors, and eventually lead to the activation of defense-related genes, cell wall strengthening, the biosynthesis of camalexin and other phytoalexins, hypersensitive response (HR)-like cell death mediated by ROS, and induced resistance against pathogens (Zhang and Zhou, 2010; Jiang et al., 2017; Kawasaki et al., 2017; Luo et al., 2017). A successful pathogen is capable of secreting and delivering effectors to the plant apoplast and cytoplasm and suppressing PTI, leading to the invasion of host. To overcome PTI impairment, plants further evolved intracellular receptors with nucleotide (NB)-Leu-rich repeat (LRR) domains that are capable of recognizing effectors and triggering ETI (Jones and Dangl, 2006). ETI is often accompanied by the accumulation of phytohormones, such as salicylic acid (SA) and a local HR-like cell death.

The plant immune system possesses several integrated signaling networks that predominantly involve protein kinases and phosphatases that perceive and respond to the different stimuli (Berriri et al., 2012; Jalmi and Sinha, 2016). One of the most important protein-kinase-based amplification cascades is the MAPK cascade. The MAPK cascade is an evolutionarily conserved signal transduction module that is involved in transducing extracellular signals to the nucleus for appropriate cellular adjustment (Li et al., 2017). The MAPK is composed of three protein kinases that sequentially activate each other by phosphorylation: a MAP kinase kinase kinase (MAPKKK) activates a MAP kinase kinase (MAPKK), which, in turn, activates a MAPK (Takahashi et al., 2007; Berriri et al., 2012). An activated MAPK phosphorylates specific substrates, such as transcription factors and enzymes and subsequently triggers cellular responses. MAPKs are activated when both tyrosine and threonine residues in the TEY/TDY motif are phosphorylated by dual-specificity kinases (MAPKKs), which, in turn, are activated by MAPKKKs *via* phosphorylation of the conserved Thr/Ser motif (Song and Goodman, 2002; Yanagawa et al., 2016). A genome-wide analysis reveals that 20 putative MAPKs, 10 MAPKKs, and 80 MAPKKKs were contained in the genome of *Arabidopsis*, with a similar repertoire of genes found in other plant species (Kong et al., 2013; Lu et al., 2015; Wang et al., 2016; Jing et al., 2017). Our previous study indicated that a total of 19 MAPKs and 5 MAPKKs were identified in the pepper genome (Liu Z. et al., 2015). Quantitative RT-PCR results revealed that *CaMAPK7* was transcriptionally upregulated by inoculation with *Ralstonia solanacearum* primarily at later time points (Liu Z. et al., 2015).

Mitogen-activated protein kinase cascades have been found to participate in the regulation of defense against biotic stresses or against the tolerance of abiotic stress, and their functions are conserved across plant species (Teardo et al., 2019; He et al., 2021). For example, *Arabidopsis* MPK3, MPK4, and MPK6 are reported to be induced by pathogen elicitors and play a vital role in innate immune responses (Takahashi et al., 2007; Berriri et al., 2012; Eschen-Lippold et al., 2016). GmMPK4 negatively regulates the accumulation of SA and defense responses of soybean against downy mildew (*Peronospora manshurica*) and soybean mosaic virus (Liu et al., 2011). The clade-A MAPKs MPK3 and MPK6 were dramatically induced by wounding and function in wounding stress (Sozen et al., 2020). A given MAPK can be involved in multiple processes. For example, *Arabidopsis* MPK4 not only participates in innate immunity against pathogens (Qiu et al., 2008; Berriri et al., 2012; Eschen-Lippold et al., 2016) but is also involved in cytokinesis and cytoskeleton organization (Beck et al., 2010, 2011; Zeng et al., 2011). However, the functions of majority of MAPK members and their possible roles in defense remain largely unknown, particularly in non-model plants.

Pepper (*Capsicum annuum*) is one of the most important vegetables worldwide. As a typical solanaceous plant, pepper frequently suffers from various soil-borne pathogens, including *R. solanacearum*, particularly when its growing environment exhibits high temperature and high humidity (Dang et al., 2013; Ali et al., 2020b). Bacterial wilt caused by the phytopathogenic bacterium *R. solanacearum* is one of the most important plant diseases worldwide (Hayward, 1991). This disease causes dramatic losses in the yield and quality of peppers, which lead to serious economic losses for pepper farmers.

Only a small number of MAPK proteins have been characterized in pepper. In pepper plants, ABA functions through CaMPK17-1-mediated MAPK signaling (Liu et al., 2016). Pepper CaMK1 and CaMK2 encode stress-inducible protein kinases that can contribute to the response to wounding, UV-C, and cold (Shin et al., 2001). Furthermore, CaMK1 and CaMK2 were found to interact with CaWRKYa and phosphorylate the SP clusters but not the MAPK docking (D) domain of CaWRKYa (Huh et al., 2015). However, the majority of MAPK kinases have not been characterized. In this study, a MAPK from the pepper genome was identified as *CaMAPK7* and functionally analyzed. The expression of *CaMAPK7* is upregulated by *R. solanacearum* infection and acts as a positive regulator in the pepper defense response against *R. solanacearum* by modifying the binding of CaWRKY40 to downstream defense-associated marker genes.

## Materials and Methods

### Plant Materials and Pathogen Inoculation

The seeds of pepper (*C. annuum* cv GZ03) and tobacco plants (*Nicotiana benthamiana* and *Nicotiana tobacum* K326) were provided by the Pepper Breeding Group of Fujian Agriculture and Forestry University, Fuzhou, China).^1^ The seeds were first germinated in sterilized ddH_2_O, and the seedlings were transferred into plastic pots and placed in the greenhouse at 25°C, 70–80 μmol photons m^–2^ s^–1^, with 70% relative humidity and a 16-h light/8-h dark photoperiod. *R. solanacearum* strain FJC100301 was isolated from wilted samples of pepper from the Fujian province (China) and cultured as described by Dang et al. (2013). For pathogen inoculation, the roots of *CaMAPK7*-silenced pepper or 6-week-old transgenic tobacco plants that ectopically expressed *CaMAPK7* were manually damaged by inserting a knife into the soil three times, and 10 ml of 10^8^ colony-forming units (CFU)/ml *R. solanacearum* FJC100301 suspended in 10 mM MgCl_2_ was infiltrated into the damaged spot using a syringe with a needle. The *R. solanacearum*-infected plants were maintained at 28°C in the greenhouse and were harvested at the indicated time points for further experiments.

### Treatment of Pepper Plants With Exogenously Applied Phytohormones and *R. solanacearum*

The leaves of 6-week-old pepper plants were sprayed with 100 μM ethylene (ET), 1 mM SA, 100 μM methyl jasmonate (MeJA), 100 μM abscisic acid (ABA), and 10 μM brassinolide (BR), respectively (Dang et al., 2013; Li et al., 2016). Pepper leaves treated with the corresponding solvent were used as a control. To study the expression pattern of *CaMAPK7* in response to infection with *R. solanacearum*, 6-week-old pepper plants were inoculated with a 10-μl suspension of FJC100301 (10^8^ CFU/ml). The phytohormone- or FJC100301-treated pepper leaves were harvested at corresponding time points for total RNA extraction and further reverse transcription assay as previously described (Liu Z. Q. et al., 2015).

### Subcellular Localization of CaMAPK7 in *N. benthamiana* Plants

The full length of *CaMAPK7* was amplified from the pepper cDNA library and cloned into the Gateway vector pMDC83 to generate 35S:*CaMAPK7-GFP* for the subcellular localization assay. GV3101 cells harboring 35S:*CaMAPK7-GFP* or 35S:*GFP* were cultured in LB media overnight, and the pellets were harvested by centrifugation. The pellets were suspended in infiltration media (10 mM MgCl_2_, 10 mM MES, pH 5.7, and 200 μm acetosyringone) and adjusted to an OD_595_ of 0.8. The suspensions were infiltrated into leaves of *N. benthamiana* plants with a needleless syringe and maintained in the greenhouse. At 48 h post-infiltration, the infiltrated leaves were harvested and subjected to fluorescent signal detection using a confocal laser scanning microscope (SP8, Leica, Wetzlar, Germany). The emission and excitation wavelengths were 488 and 510–520 nm, respectively.

### Histochemical Staining

Trypan blue staining and 3,3′-diaminobenzidine (DAB) staining were performed as described by Choi and Hwang (2011) with slight modifications (Choi and Hwang, 2011). For trypan blue staining, the pepper leaves were harvested and boiled in lactophenol/ethanol/trypan blue solution (10 ml of lactic acid, 10 ml of glycerol, 10 g of phenol, 10 ml of absolute ethanol, and 10 mg of trypan blue dissolved in 10 ml of distilled water) for 5 min and incubated at room temperature overnight. The samples were then destained in 2.5 g/ml chloral hydrate in ddH_2_O. For DAB staining, the detached pepper leaves were immersed in 1 mg/ml DAB staining buffer, incubated at 25°C overnight, and cleared with 70% ethanol. Images of pepper leaves with trypan blue and DAB staining were photographed using a dissecting microscope (Leica).

### Tobacco Transformation

Tobacco was transformed as described by Muller et al. (1984). In brief, *Agrobacterium* GV3101 cells harboring the *CaMAPK7* overexpression vector were used to infect tobacco leaf disks for transformation. Murashige and Skoog (MS) media supplemented with 500 mg/L carbenicillin and 75 mg/L hygromycin (Roche Diagnostics Corporation, Indianapolis, IN, United States^2^) was used to screen the potential tobacco transformants. The regenerated plants were further confirmed by PCR using specific primers of *CaMAPK7*. Lines #3 and #4 with a high level of expression were selected for further functional analysis.

### Chromatin Immunoprecipitation

Chromatin immunoprecipitation (ChIP) assays were performed as previously described with slight modifications (Cai et al., 2015). The full length of *CaMAPK7* was cloned into pEarleyGate 201 to generate 35S:*CaMAPK7-HA* using standard Gateway technology. *Agrobacterium* GV3101 cells harboring 35S:*CaMAPK7-HA* or 35S:*HA* (negative control) were infiltrated into 8-week-old pepper leaves. At 48 h post-infiltration, the infiltrated pepper leaves were sampled and cross-linked with 1% formaldehyde for 10 min, and 3 M glycine was added to stop the reaction and incubated for 5 min. Nuclear extracts were isolated and used for immunoprecipitation assays. Micrococcal nuclease was used to shear the isolated chromatin to a length of 200–500 bp. Anti-HA antibody was used to immunoprecipitate (IPed) the sheared chromatin, washed three times with *Tris*-buffered saline Tween 20 (TBST) buffer, and reversed cross-linked, and finally, the IPed DNA was eluted with sterile ddH_2_O. Both immunoprecipitated and input DNA were analyzed by PCR or real-time PCR with specific primers. The ChIP efficiency was calculated as percentage of input as previously described (Haring et al., 2007).

### VIGS Assay

The VIGS assay was performed as described by Liu et al. (2002) with slight modifications (Liu et al., 2002). A specific fragment of *CaMAPK7* whose specificity had been confirmed by BLASTn in the pepper genome database^3^ was amplified from the pepper cDNA library using gene-specific primers (Supplementary Table 1) and cloned into the VIGS vector TRV2 to generate TRV2:*CaMAPK7*. Transformed *Agrobacterium* cells harboring TRV1 or TRV2:*CaMAPK7* were grown, harvested, and resuspended in the infiltration medium (10 mM MgCl_2_, 10 mM MES, pH 5.7, and 200 μM acetosyringone). The resuspended *Agrobacterium* cells harboring TRV1 and TRV2 were mixed in a 1:1 ratio. At 3–4 h post-incubation at 25°C, the mixture was infiltrated into the cotyledons of pepper plants at the four-leaf stage with a needleless syringe. The *Agrobacterium*-infiltrated pepper plants were maintained in a growth chamber at 16°C for the first 56 h, and then the temperature was increased to 25°C for further growth. The *CaMAPK7*-silenced and unsilenced pepper plants were used for further experiments at 3 weeks post-agroinfiltration.

### Real-Time PCR Assay

To determine the relative transcript accumulation of target genes, real-time PCR was performed as previously described (Liu Z. et al., 2015) with specific primers (Supplementary Table 1). Briefly, the total RNA of pepper or tobacco plants was extracted using the TRIzol reagent, and DNase I was used to digest the genomic DNA in isolated RNA samples. The total RNA was reverse-transcribed using a cDNA First Strand Synthesis Kit (Vazyme, Nanjing, China) according to the manufacturer’s instructions. A Bio-Rad real-time PCR system (Bio-Rad, Hercules, CA, United States) and the SYBR premix *Ex Taq* II system were used for real-time PCR analysis. The pepper *CaActin*, *Ca18SrRNA*, or tobacco *NtEF1*α were used as internal controls. Three technical replicates were performed for at least three independent biological replicates for the real-time PCR.

## Results

### The Sequence Analysis of *CaMAPK7* and Its Promoter

**FIGURE 1:**
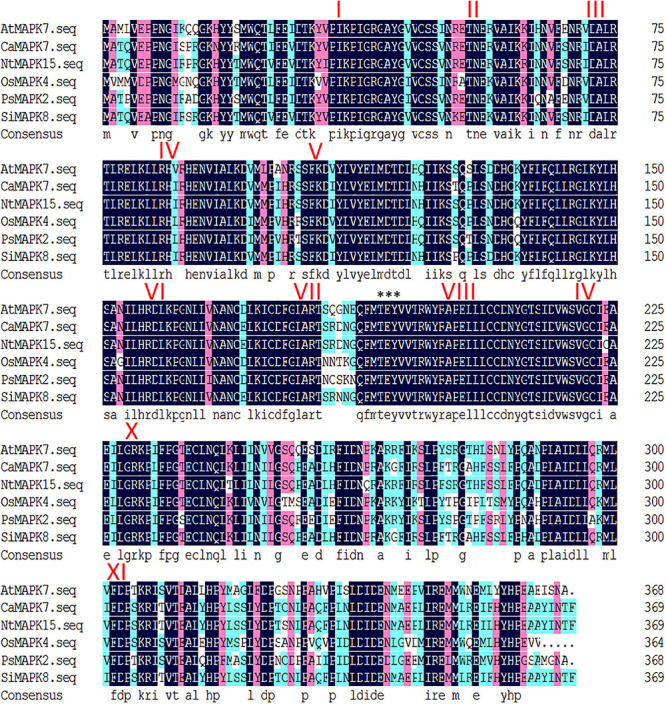
Amino acid sequence arrangements and phylogenetic analysis with the CaMAPK7 protein and its orthologs. Amino acid sequence alignments of CaMAPK7 and its orthologs from *Arabidopsis thaliana* (AtMAPK7), *Oryza sativa* (OsMAPK4), *Pisum sativum* (PsMAPK2), *Solanum lycopersicum* (SlMAPK8), and *Nicotiana tabacum* (NtMAPK15). The 11 conserved domains (I–XI) present in the serine/threonine protein kinases are denoted by Roman numerals. The conserved threonine and tyrosine residues are indicated by asterisks (***).

The genome-wide identification of pepper MAPK members was performed in our previous study, and a total of 19 MAPKs were identified in the genome of pepper variety CM334 using the pepper genome database (Liu Z. et al., 2015). Similar to other pepper MAPKs, CaMAPK7, studied herein, contains 11 domains (I–XI) that are conserved in the serine/threonine protein kinases along with a TEY motif (Figure 1). The deduced amino acid sequences of CaMAPK7 were 370 amino acid residues in length. The size and theoretical pI of the predicted protein were 42.74 kDa and 8.29, respectively. The protein sequence of CaMAPK7 and its corresponding orthologs from other species were compared and analyzed, and the results showed that CaMAPK7 shares 79.73, 80.54, 96.76, 78, and 77% of amino acid identities with AtMAPK7 (*Arabidopsis thaliana*), NtMAPK15 *(Nicotiana tabacum*), SlMAPK8 (*Solanum lycopersicum*), OsMAPK4 (*Oryza sativa*), and PsMAPK2 (*Pisum sativum*), respectively (Figure 1). In addition, the motifs contained in promoter of *CaMAPK7* was scanned using the PLANTCARE database, and various hormone- and defense-associated motifs were included, including ERE, the TCA-motif, W-box, HSE, and LTR (Supplementary Figure 1). The diverse motifs present in the *CaMAPK7* promoter indicated that CaMAPK7 may participate in the responses of pepper against biotic and abiotic stresses.

### The Expression Profile and Promoter Analysis of *CaMAPK7* Against *R. solanacearum* Infection and Exogenous Applied Phytohormones

**FIGURE 2:**
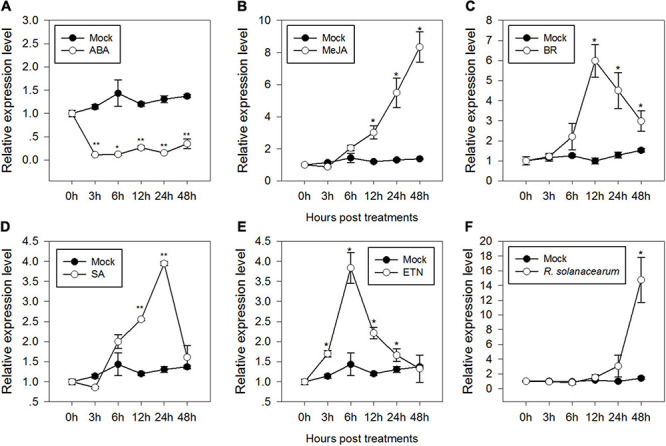
Relative *CaMAPK7* transcript levels in pepper plants in response to *Ralstonia solanacearum* infection and exogenously applied phytohormone treatments. **(A–F)** Relative *CaMAPK7* transcript levels in pepper leaves at various time periods after treatment with 100 μM ABA **(A)**, 100 μM MeJA **(B)**, 10 μM brassinolide **(C)**, 1 mM salicylic acid SA **(D)**, 100 μM ethephon **(E)**, and *R. solanacearum* **(F)**. **(A–F)** *CaMAPK7* transcript levels in pepper plants after treatment with *R. solanacearum* or hormones were compared with those in mock-treated control plants (normalized to a relative expression level of “1”). Asterisks indicate significant differences from three independent experiments based on the LSD test (*p < 0.01; **p < 0.05).

Signaling pathways mediated by phytohormones, such as ABA, JA, BR, SA, and ET, are involved in plant defense responses against biotic and abiotic stresses. To test if CaMAPK7 was involved in signaling pathways, the profile of expression of *CaMAPK7* in response to applied ABA, JA, BR, SA, and ET was studied. The results revealed that the *CaMAPK7* transcript exhibited a decrease after treatment with 100 μM ABA from 1 to 24 hpt (hours post treatment) (Figure 2A). In contrast, the relative abundance of *CaMAPK7* was enhanced after treatment with 100 μM MeJA and 1 μM BR from 12 to 48 hpt and exhibited its highest level at 48 and 12 hpt, respectively (Figures 2B,C). The exogenous SA resulted in significant increases in the accumulation of *CaMAPK7* from 12 to 24 hpt (Figure 2D) and exhibited its highest level at 24 hpt. For ethephon (ETN) treatment, the relative abundance of *CaMAPK7* began to increase at 3 hpt and lasted until 12 hpt (Figure 2E). The presence of a myriad of putative immunity-related motifs in the promoter of *CaMAPK7* implied its potential inducible expression upon pathogen attack. To confirm our hypothesis, qRT-PCR was performed to investigate the pattern of expression of *CaMAPK7* against *R. solanacearum* inoculation (RSI). The *CaMAPK7* transcript was significantly enhanced against *R. solanacearum* infection, compared with the mock-treated pepper leaves (Figure 2F).

To study the promoter activity of *CaMAPK7* in response to the applied exogenous phytohormones described above and inoculation with *R. solanacearum*, a *pCaMAPK7*-driven GUS reporter vector was generated and expressed in pepper leaves by agroinfiltration. The *Agro*-infiltrated pepper leaves were treated with phytohormones and RSI and followed by harvest for the quantification of GUS activity. GUS expression driven by *pCaMAPK7* was upregulated by JA, BR, SA, ET, and RSI, although it was downregulated by treatment with ABA (Supplementary Figure 2).

### Subcellular Localization of CaMAPK7 in *N. benthamiana* Plants

**FIGURE 3:**
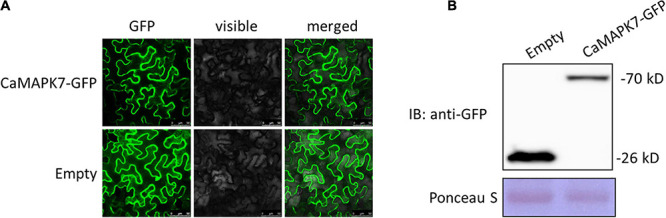
Subcellular localization of CaMAPK7 in *Nicotiana benthamiana* leaves. **(A)** CaMAPK7-GFP, similar to the empty vector, was localized throughout the whole cells. Images were taken using Leica confocal microscopy at 48 h post-agroinfiltration. Bars = 50 μm. **(B)** Immunoblot analysis of the expression of CaMAPK7 protein in *N. benthamiana* leaves. Immunoblotting used an anti-GFP antibody. Ponceau S was used to equal the loading. Empty, empty vector; GFP, green fluorescent protein; IB, immunoblotting.

The subcellular localization of a protein is closely associated with its potential function. To determine the subcellular localization of CaMAPK7, a CaMAPK7-GFP fusion construct driven by the constitutive *CaMV35S* promoter was generated and expressed in the leaves of *N. benthamiana* plant by agroinfiltration. At 48 hpt, the infiltrated leaves were harvested for the detection of fluorescence using a laser scanning confocal microscope (SP8, Leica). CaMAPK7-GFP, similar to the control GFP, was located throughout the whole plant cell, including the cytoplasm and nucleus (Figure 3A). A Western blotting assay was performed to determine the size of the CaMAPK-GFP protein (Figure 3B) expressed in the *N. benthamiana* leaves.

### Silencing of *CaMAPK7* in Pepper Plants Enhanced Its Susceptibility in Response to *R. solanacearum* Infection

**FIGURE 4:**
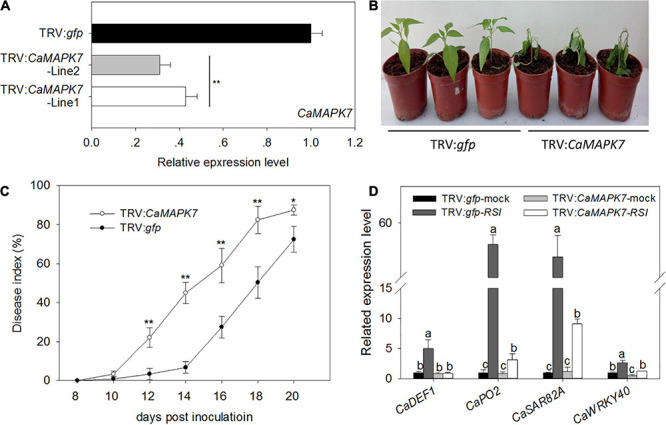
Increased susceptibility of *CaMAPK7*-silenced pepper plants to *Ralstonia solanacearum* infection. **(A)** Relative *CaMAPK7* transcript levels in empty vector control (TRV:*gfp*) and silenced (TRV:*CaMAPK7*) plants investigated by qRT-PCR. **(B)** The phenotype of *CaMAPK7*-silenced and unsilenced pepper plants 14 days post-*R. solanacearum* inoculation. **(C)** *R. solanacearum*-inoculated leaves scored every 2 days using a disease index ranging from 0 to 4: 0 (no wilting), 1 (1–25% wilted), 2 (26–50% wilted), 3 (51–75% wilted), and 4 (76–100% wilted or dead). The averages presented are based on three biological replicates each comprising 10 plants. **(A–C)** Error bars indicate the standard error. Asterisks indicate a significant difference as determined by the two-tailed *t*-test (*p < 0.05; **p < 0.01). **(D)** Real-time RT-PCR analysis of defense marker gene expression in *CaMAPK7*-silenced and unsilenced pepper plants 48 h post-inoculation with *R. solanacearum*. The relative expression of defense marker genes in unsilenced pepper leaves without pathogen inoculation was set to “1.” Data represent the means ± SD from three biological replicates. Different letters indicate significant differences in gene expression, as determined by Fisher’s protected LSD test (*p* < 0.05). dai, days after inoculation; SD, standard deviation.

To investigate the function of CaMAPK7 in pepper immunity against *R. solanacearum* infection, a loss of function of *CaMAPK7* was performed using a VIGS assay (Liu et al., 2002; Lim and Lee, 2016). The specific coding sequence (CDS) fragment of *CaMAPK7* whose specificity had been evaluated by BLASTn in NCBI was cloned into the virus vector (pTRV2) to yield pTRV2:CaMAPK7. *Agrobacterium tumefaciens* GV3101 harboring the pTRV1 vector and the pTRV2 vector with or without CaMAPK7 were mixed at a ratio of 1:1 and infiltrated into the cotyledons of pepper plants at a stage of three to four leaves. The *phytoene desaturase* gene results in a phytobleaching phenotype, and pepper plants transfected with TRV:*pds* and TRV:*gfp* were used as the positive and negative control, respectively. Each of the 80 plants of TRV:*gfp* and TRV:*CaMAPK7* was obtained and used for further experiments. Six *CaMAPK7*-silenced and unsilenced pepper plants were randomly selected for silencing efficiency detection. The transcript levels of *CaMAPK7* in *CaMAPK7*-silenced pepper plants were reduced to 25% of those in the unsilenced plants (Figure 4A), suggesting the success of silencing. *R. solanacearum* FJC100301 (Genin and Boucher, 2004) was used to infect the *CaMAPK7*-silenced and unsilenced pepper plants. Phenotypic observation showed that *CaMAPK7*-silencing enhanced the susceptibility of pepper plants in response to *R. solanacearum* infection as manifested by more obvious wilting symptoms on the TRV:*CaMAPK7* pepper plants (Figure 4B). To accurately quantify the extent of disease in *R. solanacearum*-infected plants, we determined the relative disease indices at 8–20 dpi (Figure 4C) in the TRV:*CaMAPK7* and unsilenced plants. qRT-PCR was performed to detect the transcriptional levels of defense-associated marker genes, and the accumulation of transcripts of the defense-related marker genes, including *CaDEF1*, *CaPO2*, *CaSAR82A*, and *CaWRKY40*, was lessened in FJC100301-infected *CaMAPK7*-silenced pepper plants at 24 hpi, compared with the unsilenced pepper plants (Figure 4D).

### Transient Overexpression of *CaMAPK7* Elicits Intensive HR-Like Cell Death and the Accumulation of H_2_O_2_ in Pepper Leaves

**FIGURE 5:**
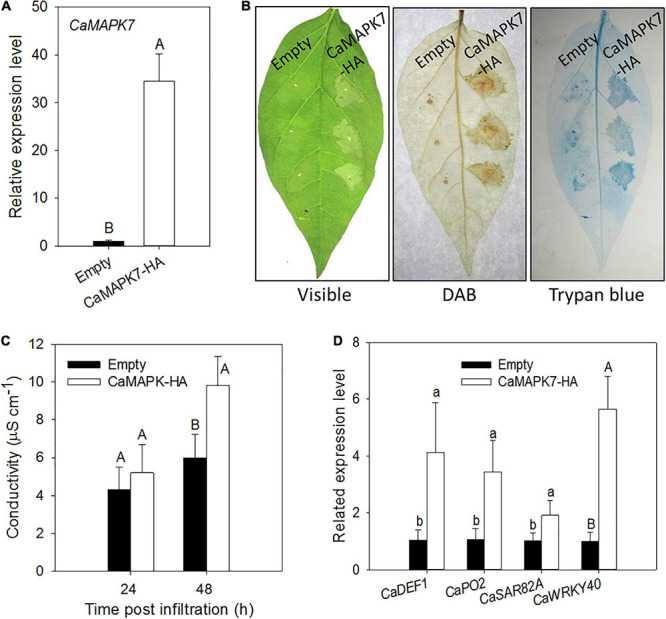
Effects of the transient expression of *CaMAPK7* on the cell death response of pepper leaves. **(A)** qRT-PCR analysis of transiently expressed *CaMAPK7* in pepper leaves at 24 h post-agroinfiltration. **(B)** Induction of cell death and H_2_O_2_ accumulation by transient *CaMAPK7* expression in pepper leaves. The images of phenotype (left), trypan blue staining (middle), and DAB staining (right) were photographed 2 days post-agroinfiltration. **(C)** Electrolyte leakage from pepper leaves transiently expressing *CaMAPK7* at 24 and 48 h after inoculation. **(D)** Real-time analysis of defense-associated marker genes in *CaMAPK7*-transient expression pepper leaves. The relative expression level of defense marker genes in pepper plants transiently expressed empty vector were set to “1.” **(C,D)** Error bars indicate the standard error. Different letters above the bar show a significant difference between the means of the three biological replicates based on two-tailed *t*-test: uppercase letters, *p* < 0.01; lowercase letters, *p* < 0.05. DAB, diaminobenzidine.

The *CaMAPK7*-silencing experiment in pepper plants indicates that CaMAPK7 acts as a positive regulator in pepper immunity in response to *R. solanacearum* infection. To further confirm our hypothesis, a transient overexpression assay was conducted to study the effect of *CaMAPK7* transient overexpression on the induction of HR-like cell death in pepper leaves. GV3101 cells that harbored *CaMAPK7-HA* were infiltrated into the leaves of pepper plants for transient overexpression. The qRT-PCR results showed that *CaMAPK7-HA* was successfully overexpressed in pepper leaves (Figure 5A). An intensive HR-like cell death was observed in pepper leaves that transiently overexpressed *CaMAPK7-HA*, manifested by darker trypan blue staining and higher electrolyte leakage release. In addition, *CaMAPK7* transient overexpression triggered obvious H_2_O_2_ accumulation, confirmed by DAB staining (Figures 5B,C). qRT-PCR was further performed to examine the transcript accumulation of defense-associated marker genes, and the overexpression of *CaMAPK7* could elicit the upregulation of many marker genes, including *CaDEF1*, *CaPO2*, and *CaSAR82A* (Figure 5D). Interestingly, we found that the overexpression of *CaMAPK7* can also induce the transcriptional level of *CaWRKY40* (Figure 5D), a transcription factor reported in our previous study that plays a vital role in the immunity of pepper against *R. solanacearum* (Dang et al., 2013).

### Ectopic Overexpression of *CaMAPK7* in Tobacco Plants Enhanced Disease Resistance Against *R. solanacearum* Inoculation

**FIGURE 6:**
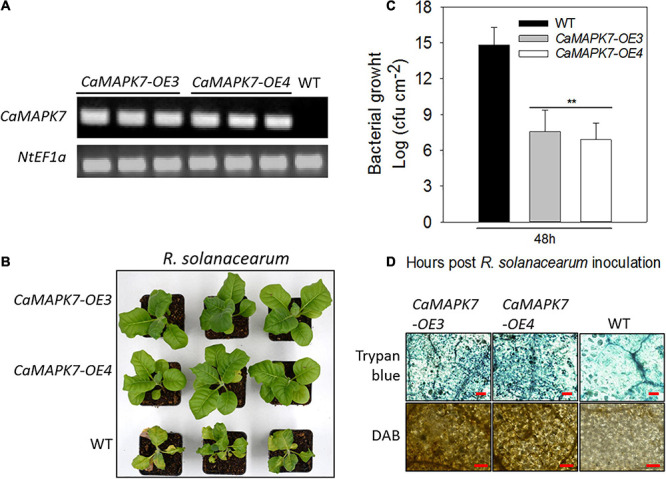
Transgenic T3 tobacco plants ectopically overexpressing *CaMAPK7* exhibit enhanced resistance to *Ralstonia solanacearum*. **(A)** Transcript accumulation of *CaMAPK7* in CaMAPK7-OX lines (#3 and #4) and WT detected by semi-quantitative PCR. **(B)** The disease phenotype of CaMAPK7-OX lines and WT 10 dpi with *R. solanacearum* strain FJC100301 in the plant roots. **(C)** The growth of *R. solanacearum* in leaves of CaMAPK7-OX lines and WT at 48 h post-*R. solanacearum* inoculation. Values are average CFU based on three biological replicates. Asterisk indicates significant differences determined by the Fisher’s protected LSD test (**p < 0.01). **(D)** HR-like cell death and H_2_O_2_ accumulation detected in CaMAPK7-OX lines and WT induced by *R. solanacearum* infection. Bar = 500 μm. dpi, days post-inoculation; HR, hypersensitive response; WT, wild-type.

To further confirm the results of VIGS and transient overexpression, transgenic tobacco lines that ectopically overexpressed *CaMAPK7* driven by two copies of the *CaMV35S* promoter were generated. More than 10 T3 lines were acquired, and two lines of 10 (lines 3 and 4) were selected for further functional analysis. Semi-quantitative RT-PCR was performed to detect the *CaMAPK7* transcript in CaMAPK7-OX-3, CaMAPK7-OX-4, and wild-type (WT) tobacco plants (Figure 6A). Transgenic tobacco plants and the WT were subjected to *R. solanacearum* infection. At 8 dpi, an obvious wilting symptom was observed in the WT tobacco plants, whereas the CaMAPK7-OX lines only wilted slightly (Figure 6B). The CFU of *R. solanacearum* in the CaMAPK7-OX line and WT were calculated at 3 dpi, and the ectopic expression of *CaMAPK7* in tobacco plants significantly suppressed the growth of *R. solanacearum*, compared with that in the WT tobacco plants (Figure 6C). Trypan blue and DAB staining were performed to evaluate the local defense response in CaMAPK7-OX and WT tobacco plants. *R. solanacearum* infection in the CaMAPK7-OX lines elicited more severe HR-like cell death and H_2_O_2_ accumulation than that in WT tobacco plants (Figure 6D).

**FIGURE 7:**
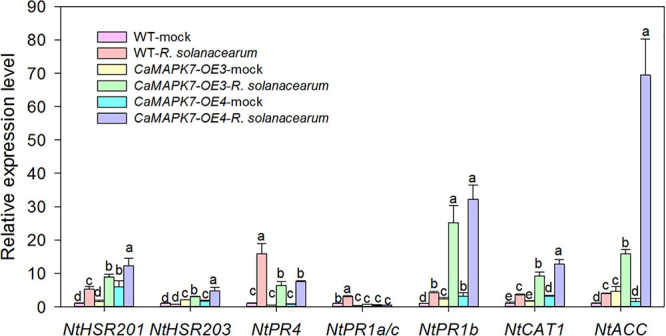
qRT-PCR analyses of relative expression levels of defense marker genes in CaMAPK7-OX lines compared with WT tobacco plants. Defense-related transcript accumulation of WT tobacco plants were used as reference, which was set to “1.” Different letters above the bar show a significant difference between the means of the three biological replicates based on Fisher’s protected LSD test: lowercase letters, *p* < 0.05. WT, wild-type.

To further confirm the role of *CaMAPK7* in disease resistance and investigate its possible molecular mode of action, the transcriptional response of well-known defense-associated marker genes in the CaMAPK7-OX lines and WT tobacco plants were detected using qRT-PCR. We examined the accumulation of transcripts of the HR-associated genes *NtHSR201* and *NtHSR203*, JA-responsive genes *NtPR1b* and *NtPR4*, SA-responsive genes *NtPR1a/c*, ROS-associated *NtCAT1*, and the ethylene-related gene *NtACC*. All the marker genes tested in the CaMAPK7-OE lines above were shown to be significantly higher than those in WT tobacco plants (Figure 7).

### The Effect of *CaMAPK7* Transient Overexpression on the Binding of CaWRKY40 to Its Target Genes

**FIGURE 8:**
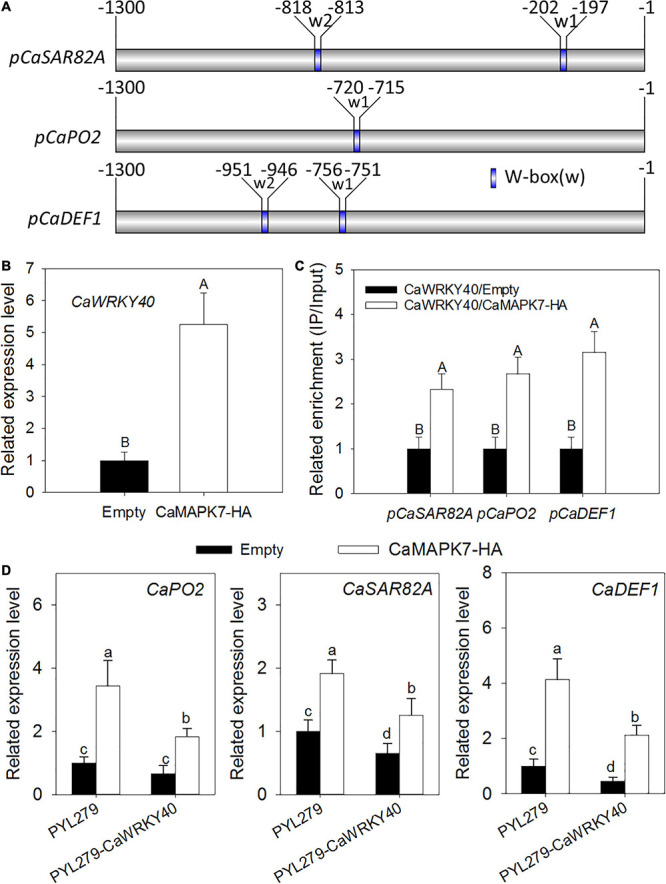
The transcriptional expression of *CaWRKY40* triggered by *CaMAPK7* overexpression and the bindings of CaWRKY40 to the promoters of its target genes were enhanced by *CaMAPK7* expression. **(A)** Schematic representation of a typical W-box in the promoters of CaWRKY40 downstream defense marker genes. **(B)** *CaWRKY40* transcript level triggered by CaMAPK7 overexpression in pepper leaves. The *CaWRKY40* transcript level in pepper leaves transient expressed empty vector was set to “1.” **(C)** The binding of CaWRKY40 to the promoters of its target genes was enhanced by *CaMAPK7* transient expression in pepper plants. Relative enrichment levels of samples of the *CaWRKY40* transient overexpression were set to “1” after normalization by input. **(D)** Real-time PCR analysis of the defense-associated marker genes in *CaWRKY40*-silenced pepper leaves transiently overexpressing *CaMAPK7*. The expression of defense-related marker genes in unsilenced pepper leaves transiently expressing vector was set to “1.” **(B–D)** Data are the means ± SD from at least three independent experiments. Different letters above the bar show a significant difference between the means of the three biological replicates based on Fisher’s protected LSD test: uppercase letters, *p* < 0.01; lowercase letters, *p* < 0.05. SD, standard deviation.

The transient overexpression assay revealed that *CaMAPK7* overexpression triggered the upregulation of CaWRKY40, a WRKY transcription factor involved in pepper immunity against *R. solanacearum*; moreover, one or two W-box elements were found to be contained in the promoters of the defense-related marker genes *CaSAR82A*, *CaPO2*, and *CaDEF1* regulated by CaMAPK7 (Figures 8A,B). We hypothesize that CaMAPK7 regulates the expression of marker genes above *via* the modification of the binding of CaWRKY40 to the promoter of marker genes described above. ChIP and PCR assays were performed to verify our hypothesis. To conduct this experiment, GV3101 cells harboring *CaMAPK7-HA* or an empty vector were infiltrated into the leaves of 6-week-old pepper plants and maintained in the greenhouse. At 48 hpi, the infiltrated pepper leaves were harvested and cross-linked with 1% formaldehyde, and the chromatin was isolated as described in our previous study (Shen et al., 2016a) with slight modifications. Primers flanking the W-box of the marker genes were used for ChIP-PCR. For promoters with more than one W-box, the primer pairs were screened for product amplification and used in the real-time PCR measurements of specific CaWRKY40 binding to the promoter. The binding of CaWRKY40 to the promoters of *CaSAR82A*, *CaPO2*, and *CaDEF1* was significantly enhanced by the overexpression of *CaMAPK7* (Figure 8C). *CaWRKY40*-silenced pepper plants were acquired to study the effect of *CaWRKY40*-silencing on the regulation of CaMAPK7 on downstream marker genes described above. VIGS and qRT-PCR assays showed that in the unsilenced pepper plants, *CaMAPK7* overexpression significantly increased the expression of defense-marker genes, including *CaPO2*, *CaDEF1*, and *CaSAR82A*, whereas the increase was partially abolished in *CaWRKY40*-silenced pepper plants (Figure 8D).

## Discussion

### CaMAPK7 Is a Positive Regulator of Defense Responses and Plant Cell Death in Pepper Plants

At the extreme downstream of MAPKs cascade, MAPKs were reported to play vital roles in plant growth, development, and responses to several environmental challenges *via* the phosphorylation of a target substrate. However, most of the studies of MAPKs have focused on model plants, such as *Arabidopsis* and rice. Therefore, the roles of MAPK in non-model plants such as pepper remain to be elucidated. The most extensively studied plant MAPKs are *Arabidopsis* AtMPK6, AtMPK3, and AtMPK4, all of which are activated by several stimuli, including biotic and abiotic stresses (Ichimura et al., 2000; Droillard et al., 2002; Pitzschke et al., 2009; Schikora et al., 2011; Dutilleul et al., 2012). Nineteen MAPKs were identified in the pepper genome in our previous study, although only 2 MAPKs out of 19, MK1 and MK2 (designated CaMPK3 and CaMPK6-1 in our previous study), were partially functionally analyzed (Shin et al., 2001). MK1 and MK2 encode stress-inducible protein kinases that can contribute to the response to wounding, UV-C and cold. The identification and functional analysis of other MAPK members in pepper plants will benefit from the dissection of MAPK cascades in pepper. In this study, a novel pepper MAPK *CaMAPK7* was identified, and its function in response to *R. solanacearum* was analyzed. Pathogen infection induces the expression of many MAPK genes (Lumbreras et al., 2010). Of the 19 pepper MAPK genes, 12 were found to be differentially regulated following treatment with *R. solanacearum* (Liu Z. et al., 2015). We found *CaMAPK7* transcripts to be induced by inoculation with *R. solanacearum*. Gene expression is strictly regulated by the upstream promoter, and the motifs contained in the promoter were associated with the potential functions of target gene. The results analyzed on PLANTCARE showed that diverse motifs, including stress-related motifs, were contained in the *CaMAPK7* promoter, including the transcription factor WRKY binding motif W-box (4), ethylene-responsive ERE box (1), transcription factor MYB binding motif MBS (1), low-temperature-responsive element LTR (1), and heat shock element HSE (1). These *cis*-elements have been reported to be involved with the plant signaling pathway and immunity responses. We further investigated the activities of the *CaMAPK7* promoter (*pCaMAPK7*), and the data showed that *pCaMAPK7* significantly induces GUS activities in response to infection with *R. solanacearum* (Supplementary Figure 1). The overexpression of CaMAPK7 enhanced resistance of transgenic tobacco to inoculation with *R. solanacearum*, suggesting a positive contribution of CaMAPK7 to resistance against bacterial pathogens. Consistent with this, the resistance of pepper plants to inoculation with *R. solanacearum* was attenuated in *CaMAPK7*-silenced pepper plants. In *Arabidopsis*, MPK7 was activated by upstream MKK3 in response to ABA, but poorly activated by flagellin-derived flg22 peptide treatment, suggesting that MPK7 participates in ABA signaling pathway and plays a negative role in PTI.

A typical event of a defense response is the accumulation of transcripts of HR-associated and pathogen-responsive (*PR*) genes and usually serves as a marker for the activation of plant immunity. In tobacco, the expression of *NtHSR201* and *NtHSR203* is frequently upregulated during defense responses against bacterial pathogens (Czernic et al., 1996; Pontier et al., 1998; Lee et al., 2004). The overexpression of *CaMAPK7* in transgenic tobacco constitutively upregulates the expression of *NtHSR201* and *NtHSR203*. The immunity mediated by CaMAPK7 against *R. solanacearum* is likely based on the prompting effect of *CaMAPK7* on transcription of the HR-associated and *PR* genes. Furthermore, the transient overexpression of CaMAPK7 in pepper leaves triggers insensitive HR-like cell death accompanied with a significant burst of ROS. It is worth noting that *NtHSR203*, a negative regulator of plant hypersensitive cell death (Pontier et al., 1998; Tronchet et al., 2001), was upregulated in tobacco plants by *R. solanacearum* infection and also by *CaMAPK7* overexpression in tobacco plants uninoculated or inoculated with *R. solanacearum*. This finding that *NtHSR203* was upregulated in transgenic tobacco plants that ectopically overexpressed *CaWRKY40* during the defense response of pepper against *R. solanacearum* was also observed in our previous study (Dang et al., 2013). We hypothesize that the upregulation of *NtHSR203* triggered by *R. solanacearum* and *CaMAPK7* overexpression serves to avoid the inappropriate activation of defense responses. Collectively, these results suggest that CaMAPK7 functions as a positive regulator of defense response and in HR-like cell death in pepper leaves.

### CaMAPK7 May Play a Vital Role in Defense Signaling Mediated by SA, JA, ET, and BR

The production of SA, JA, ET, and BR is frequently induced in response to various pathogens. The balance of these hormones is indispensable in fine-tuning appropriate defense responses (Dong, 1998; Dangl and Jones, 2001; Divi et al., 2010). SA, JA, ET, and BR have been reported to activate numerous plant *PR* genes and act synergistically or antagonistically during defense signaling dependent on their concentrations (Bari and Jones, 2009; Yang et al., 2015, 2019; Pan et al., 2018). We found *CaMAPK7* transcripts to be induced in response to the exogenous application of SA, JA, ET, and BR. The overexpression of *CaMAPK7* in pepper leaves led to the upregulation of JA-responsive *CaDEF1*, SA-associated *CaSAR82A*, and ROS-related *CaPO2*. In addition, the overexpression of CaMAPK7 in transgenic tobacco enhanced the levels of transcripts of SA-responsive *NtPR1a/c*, JA-responsive *NtPR4* and *NtPR-b*, the ROS detoxification-associated gene *NtCAT1*, and the ethylene production-associated *NtACC* in response to inoculation with *R. solanacearum*. It is worth noting that ABA treatment decreased the level of transcript of *CaMAPK7*, which supports an antagonistic relationship between ABA and SA in host–pathogen interactions (Nahar et al., 2012). Therefore, we hypothesize that SA, JA, ET, and BR regulate the expression of CaMAPK7, leading to the expression of downstream defense-associated marker genes.

### *CaMAPK7* Transient Overexpression Modifies the Binding of CaWRKY40 to Its Target Genes

**FIGURE 9:**
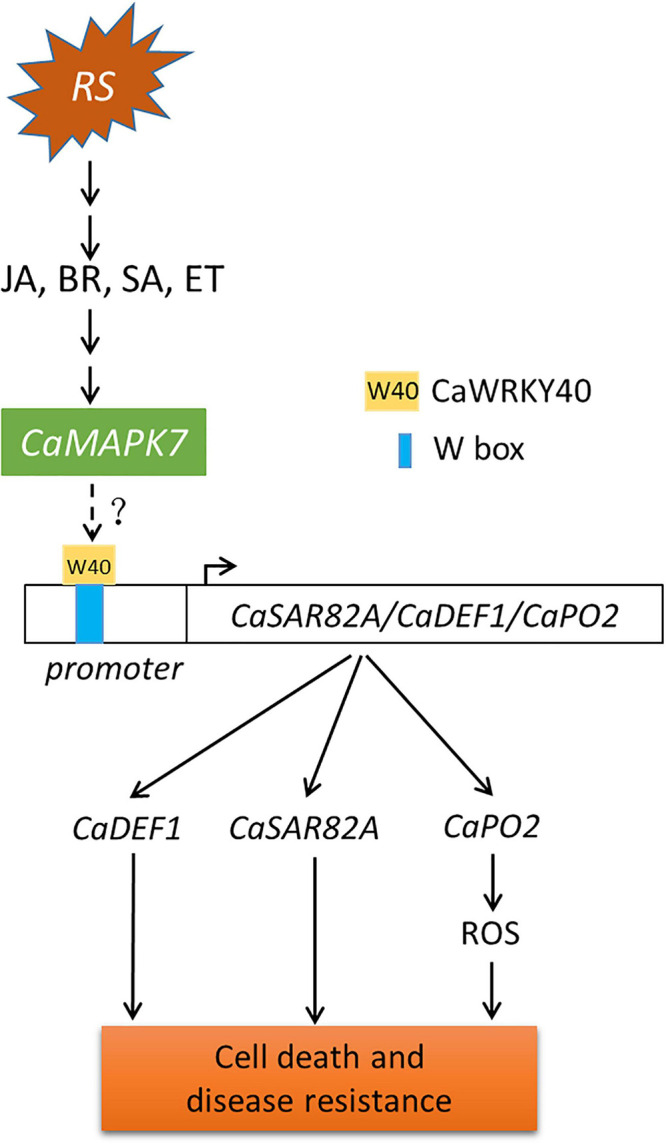
Proposed model of CaMAPK7 in response to inoculation with *Ralstonia solanacearum* in pepper plants. The pepper CaMAPK7 has a positive effect on the trans-activation of CaWRKY40, which regulates the expression of defense-associated marker genes, ultimately leading to cell death and disease resistance. Arrows indicate positive regulation, and dotted ends denote unknown regulation. RS, *Ralstonia solanacearum*.

Although MAPKs and WRKYs are both reported to participate in plant immune response to pathogens, the molecular linkage between these two proteins has not been fully established. Our previous study indicated that the *CaWRKY40* transcript was upregulated by inoculation with *R. solanacearum* and acts as a positive regulator of pepper immunity against *R. solanacearum*. The fact that *CaMAPK7* and *CaWRKY40* exhibited similar patterns of expression in response to *R. solanacearum* and applied exogenous hormones, including SA, MeJA, and ET, implies a potential relationship between these two proteins. This hypothesis was supported by the result that the *CaWRKY40* transcript was significantly induced by the overexpression of *CaMAPK7* and downregulated in *CaMAPK7*-silenced pepper plants, compared with the unsilenced pepper plants. CaMAPK7, located in the plasma membrane and cytoplasm, is believed to be involved in the early signaling pathway and participates in pepper defense response against *R. solanacearum* infection. CaWRKY40, activated by upstream CaWRKY6 and CaWRKY40b in our previous study (Cai et al., 2015; Ifnan Khan et al., 2018), is expected to function in *R. solanacearum*-induced pepper immunity *via* regulation of the downstream target genes, such as *CaC3H14*, *CabZIP63*, and *CaCDPK15* (Shen et al., 2016a, b; Qiu et al., 2018). Of note is that CabZIP63 and CaCDPK15 directly or indirectly, respectively, regulate the expression of CaWRKY40 at both the transcriptional and post-transcriptional level, forming a positive feedback loop with CaWRKY40 during the response of pepper to RSI or high temperature and high humidity (Shen et al., 2016a, b). Similar molecular linkages between MAPKs and WRKYs have been reported previously. For example, a study by Adachi et al. (2015) showed that four WRKY transcription factors were phosphorylated by MEK2 to regulate a plant immune NADPH oxidase in *N. benthamiana* (Adachi et al., 2015). Ishihama et al. (2011) provided evidence that the *N. benthamiana* WRKY8 transcription factor is physiologically phosphorylated by SIPK, NTF4, and WIPK, and the phosphorylation of WRKY8 increased its DNA binding activity to the cognate W-box sequence (Ishihama et al., 2011) and triggered the expression of downstream genes. Furthermore, the VIGS and qRT-PCR assays in this study showed that in unsilenced pepper plants, the overexpression of *CaMAPK7* significantly increased the expression of defense-marker genes, including *CaPO2*, *CaDEF1*, and *CaSAR82A*, whereas the increase was partially abolished in *CaWRKY40*-silenced pepper plants. Based on the results described above and our previous study, we hypothesize that CaMAPK7 may regulate the expression of defense-related marker genes, *CaDEF1*, *CaPO2*, and *CaSAR82A*, in a CaWRKY40-dependent manner. In addition, the inducible expression of *CaWRKY40* in response to *R. solanacearum* was significantly attenuated in *CaMAPK7*-silenced pepper plants, suggesting that CaWRKY40 functions in pepper defense against *R. solanacearum* probably in a CaMAPK7-dependent manner. We hypothesize that CaMAPK7 may interact with CaWRKY40 and directly modify the regulation of CaWRKY40 on downstream marker genes. Unexpectedly, our co-IP assay showed that no binding was observed between CaMAPK7 and CaWRKY40 (data not shown). One explanation is that CaMAPK7 might phosphorylate and activate other component that directly targets CaWRKY40, and the phosphorylated component acts as a bridge between CaMAPK7 and CaWRKY40. Further identification of possible CaMAPK7 interactors that subsequently target CaWRKY40 could provide new insights into the mechanism of pepper immunity mediated by CaMAPK7 and CaWRKY40. Taken together, we propose a working model that infection with *R. solanacearum* induces the accumulation of SA, JA, BR, and ET and activates the expression of CaMAPK7, which subsequently modifies the transcriptional activity of CaWRKY40, ultimately leading to cell death and disease resistance in pepper plants (Figure 9).

In summary, our data demonstrate that CaMAPK7 responds to infection with *R. solanacearum* and plays a positive role in resistance to *R. solanacearum* by modifying the trans-activation of CaWRKY40. These results suggest a possible molecular mechanism involving CaMAPK7 in *R. solanacearum* responses and provides insight into the MAPK cascade in pepper plants.

## Data Availability Statement

The raw data supporting the conclusions of this article will be made available by the authors, without undue reservation.

## Author Contributions

LS, SH, and ZL designed the experiments. LS, KZ, LX, MY, and BX performed the experiments and the data analyses. LS, SH, and ZL prepared the figures and wrote the manuscript. All authors contributed to the article and approved the submitted version.

## Conflict of Interest

The authors declare that the research was conducted in the absence of any commercial or financial relationships that could be construed as a potential conflict of interest.

## Footnotes

1 www.fafu.edu.cn

2 http://www.roche.com

3 http://passport.pepper.snu.ac.kr/?t=PGENOME

## Supplementary Material

Supplementary Figure 1 The distribution of potential motifs in *CaMAPK7* promoter CAATBOX, *cis*-acting regulatory element involved in light responsiveness; W-box, WRKY transcription factor binding site; ERE, ethylene-responsive element; TCA, *cis*-acting regulatory element involved in salicylic acid responsiveness; ARE, *cis*-acting regulatory elements essential for the anaerobic induction; MBS, MYB binding site; HSE, *cis*-acting regulatory element involved in heat stress responsiveness; LTR, *cis*-acting element involved in low-temperature responsiveness; TATA-Box, core promoter of the transcription start site.

Supplementary Figure 2 Relative GUS activities driven by the *CaMAPK7* promoter in transgenic tobacco plants in response to *Ralstonia solanacearum* inoculation and the application of exogenous phytohormones. **(A–F)** Relative GUS activities in tobacco plants harboring *pCaMAPK7:GUS* at various time periods after treatment with 100 μM ABA **(A)**, 100 μM MeJA **(B)**, 10 μM Brassinolide **(C)**, mM SA **(D)**, 100 μM ET **(E)**, 1 and *R. solanacearum* **(F)**. The GUS activity of transgenic tobacco plants carrying *CaMAPK7*:GUS without treatments were set to “1.” Error bars indicate the standard error. Different letters above the bar show a significant difference between the means of three biological replicates based on Fisher’s protected LSD test: uppercase letters, *p* < 0.01; lowercase letters, *p* < 0.05.

Supplementary Table 1 Oligonucleotides for the plasmid constructs and PCR used in this study.
