# Supplementary material for: The Pepper Mitogen-Activated Protein Kinase CaMAPK7 Acts as a Positive Regulator in Response to Ralstonia solanacearum Infection
Source: Front Microbiol. 2021 Jul 6;12:664926. doi: 10.3389/fmicb.2021.664926 (PMC8290481; doi:10.3389/fmicb.2021.664926)
Supplement: Supplementary Figure 1 — The distribution of potential motifs in CaMAPK7 promoter CAATBOX, cis-acting regulatory element involved in light responsiveness; W-box, WRKY transcription factor binding site; ERE, ethylene-responsive element; TCA, cis-acting regulatory element involved in salicylic acid responsiveness; ARE, cis-acting regulatory elements essential for the anaerobic induction; MBS, MYB binding site; HSE, cis-acting regulatory element involved in heat stress responsiveness; LTR, cis-acting element involved in low-temperature responsiveness; TATA-Box, core promoter of the transcription start site. [file Data_Sheet_1.docx]

**Figure S1 The distribution of potential motifs in *CaMAPK7* promoter.** CAATBOX: *cis*-acting regulatory element involved in light responsiveness; W-box: WRKY transcription factor binding site; ERE: ethylene-responsive element; TCA: *cis*-acting regulatory element involved in salicylic acid responsiveness; ARE: *cis*-acting regulatory elements essential for the anaerobic induction; MBS: MYB binding site; HSE: *cis*-acting regulatory element involved in heat stress responsiveness; LTR: *cis*-acting element involved in low-temperature responsiveness; TATA-Box: core promoter of the transcription start site.

**Figure S2 Relative GUS activities driven by *CaMAPK7* promoter in transgenic tobacco plants in response to *R.* *solanacearum* inoculation and applied exogenous phytohormones.** (A-F) Relative GUS activities in tobacco plants harboring *pCaMAPK7:GUS* at various time periods after treatment with 100 µM ABA (A), 100 µM MeJA (B),10 µM Brassinolide（C）, mM SA (D),100 µM ET (E), 1 and *R.* *solanacearum* (F).The GUS activity of transgenic tobacco plants carrying CaMAPK7:GUS without treatments were set to “1”. Error bars indicate the standard error. Different letters above the bar show a significant difference between the means of the three biological replicates based on the Fisher’s protected LSD test: uppercase letters, *p* < 0.01; lower case letters, *p* < 0.05.
